# Exploring Determinants of Early Retirement Among Saudi Medical Staff

**DOI:** 10.3389/fpsyg.2021.743393

**Published:** 2021-12-23

**Authors:** Mohammed A. AlKhars, Taqi N. AlFaraj, Ahmad H. AlNasser

**Affiliations:** KFUPM Business School, King Fahd University of Petroleum and Minerals, Dhahran, Saudi Arabia

**Keywords:** early retirement, push and pull theory, medical staff, Saudi Arabia, gender difference

## Abstract

The aim of this research was to explore the relationship between the push, pull, anti-push, and anti-pull factors vs. early retirement intention among Saudi medical staff, and to investigate whether there are gender differences in the early retirement intention. To this end, we designed a correlational and cross-sectional study, for which data were collected through an online survey. A total of 680 responses were gathered, of which 221 valid responses constituted the final sample for the analysis. Logistics regression was used to test the hypotheses of the study. The results showed that approximately 58% of the respondents indicated early retirement intention. The significant factors in predicting this intention were the pull, anti-push, and anti-pull factors, whereas the push factors were found to be insignificant. Moreover, female medical staff tend to retire earlier than males. Strategies recommended to delay retirement are providing flexible work hours, working shorter shifts or on a part-time basis, offering programs for professional development, and according more recognition.

## Introduction

Unlike many developed countries that face the issue of an aging population, which threatens the labor market and influences the ability to maintain an affordable social security system (Blakeley and Ribeiro, 2008; Sejbaek et al., 2013; Duffield et al., 2015; Fouquereau et al., 2018; Wargo-Sugleris et al., 2018; Hewko et al., 2019), the problem faced by Saudi Arabia is an instability of medical manpower. Approximately 47% of the medical staff are expatriates, who usually work on short temporary contracts to reimburse shortages in the national medical labor force (Saudi Ministry of Health, 2020). Many national medical professionals, who account for 53% of the entire medical staff, retire early. The existence of temporary expatriates causes a great cash outflow, which negatively impacts the national economy. The low percentage of nationals underlines the importance of encouraging existing national medical staff to avoid early retirement and extend their service. This step will positively affect the sustainability of the country’s medical services. To encourage work continuation among existing senior medical staff, more knowledge about the factors that influence early retirement is needed.

Several factors related to job environment, such as stress and unpleasant atmosphere, could affect the well-being of employees and lead them to early retirement. Many theories in the literature are used to predict employee well-being, such as the demand–control model (Karasek, 1979), the effort–reward imbalance model (Siegrist, 1996), and the job demands–resources model (Bakker and Demerouti, 2007). The demand–control model (Karasek, 1979) states that the disequilibrium between job demand and recourses at hand could cause job strain. Thus, high job demands, such as work overload and time pressure, accompanied by low job control, such as controlling the task at hand, result in job strain. On the contrary, the effort–reward imbalance model (Siegrist, 1996) avers that the imbalance between efforts and rewards rather than control is the cause of job strain. Thus, high efforts, such as high job demands, and low rewards, such as low salary, low esteem rewards, or low career opportunities, can cause job strain. The final model is the job demands–resources model (Bakker and Demerouti, 2007), which holds that every occupation may have its own risk factors. These can be grouped into two categories: job demands and job resources. The former could be physical, psychological, social, or organizational aspects requiring efforts that could cause stress in the job. Job resources are those physical, psychological, social, or organizational aspects that could help achieve job goals or reduce job demands as well as related psychological and physiological costs. Despite the importance of these models in identifying the factors predicting the well-being of employees, a specific theory is needed to account for the factors that predict the intention of employees to retire early. This study used the push, pull, anti-push, and anti-pull factors as a framework (Fouquereau et al., 2018) to explore the early retirement of medical staff in Saudi Arabia.

This study aims to gain insights regarding the Saudi medical staff intentions and explore their considerations of early retirement or extension of service. The official retirement age in Saudi Arabia is 60 years for both men and women. Saudi employees can continue working beyond this age (until 65, 70, or over) in exceptional cases, for which they must obtain certain approvals. Saudis are eligible to retire early after at least 15 years of service, but they will receive only a lump-sum one-time payment and not a monthly retirement pension. However, after completing 20 years of service and obtaining approval from employers, Saudi employees are eligible for a monthly pension payment (Saudi General Organization for Social Insurance, 2020). The outcomes of this study could help administrators and policy makers develop incentives to delay the retirement of the Saudi medical staff and encourage them to continue working. The research questions addressed in this study were as follows:

1. What are the retirement intentions of senior medical staff?
2. What are the significant factors that senior medical staff consider when intending to retire early?
3. Is there a difference between female and male medical staff in terms of the intention to retire early?

To the best of our knowledge, there is only one published work on early retirement in Saudi Arabia, that examined the moderating roles of age and organizational culture on the relationship between early retirement intention and its factors within the industrial sector (Al-Jumaah and Ashari, 2017). There is no published work on the factors of early retirement among Saudi medical staff. This study intends to fill this part of this gap.

The paper is organized as follows. The next section presents a brief literature review followed by an explanation of the methodology and data analysis. A general discussion is then provided. Finally, the paper concludes with policy implications, limitations, and future research.

## Literature Review

Determinants of early retirement decisions vary among individuals in different countries, sectors, and occupations. Researchers used different classifications to meet the objectives of their studies. For example, Wang and Shultz (2010) presented a review of the issues and relationships of published empirical studies on the retirement process. They used four main categories: individual attributes, job and organizational factors, family factors, and socio-economic factors. Furthermore, Blakeley and Ribeiro (2008) classified the factors into three categories: personal, financial, and work-related. In another study, early retirement factors were classified into individual, work-related, and organizational factors (Boumans et al., 2008). Similarly, a recent study conducted a meta-analysis of early retirement antecedents and subsequent correlates (Topa et al., 2017). They classified the antecedents into four levels: individual, job, work, and family. Earlier, Shultz et al. (1998) classified early retirement factors into push and pull factors. Later, Fouquereau et al. (2018) added two more dimensions: anti-push and anti-pull factors. This latest classification of push, pull, anti-push, and anti-pull will be used as a framework to investigate the early retirement factors within the context of the medical sector in Saudi Arabia.

Mullet et al. (2000) were the first to use the push, pull, anti-push, and anti-pull framework to study the decision-making process of French youths to study, work, and live in another European country. Fouquereau et al. (2018) utilized this framework and created a questionnaire to study the psychological decision-making process of retirement. Push factors are negative considerations, such as poor health, which induce older workers to retire early (Fouquereau et al., 2018). Conversely, pull factors are positive considerations, such as the desire to relax after retirement, that cause people to retire early (Fouquereau et al., 2018). Anti-push factors are defined as the attachment to the present situation, such as playing an active role at work, while anti-pull factors are the perceived costs and risks of future situations, such as feeling depressed after retirement (Fouquereau et al., 2018). It is worth noting that push and anti-push factors deal with the present situation, while the pull and anti-pull factors deal with the future state. The following paragraphs briefly review the literature on the factors that affect early retirement decisions among medical staff using the aforementioned framework.

Poor health is one of the main push factors that affects early retirement decisions. It is significant among physicians, nurses, and hospital employees (Boumans et al., 2008; Heponiemi et al., 2008; Suadicani et al., 2013; Duffield et al., 2015; de Oliveira et al., 2017). Similarly, sickness absences is a crucial factor in predicting early retirement decisions (Heponiemi et al., 2008; Suadicani et al., 2013; de Oliveira et al., 2017). Being tired of work is found to be another decisive factor among Canadian registered nurses (Blakeley and Ribeiro, 2008; Hewko et al., 2019), but is not noteworthy among allied health professionals (Hewko et al., 2019). High work demand, quantitative demands, and heavy workloads are vital factors (Blakeley and Ribeiro, 2008; Boumans et al., 2008; Sejbaek et al., 2013; de Oliveira et al., 2017). However, Suadicani et al. (2013) found that high work demand was not significant in the early retirement decision among Danish hospital employees. Low job control is important in predicting early retirement among Finnish physicians (Heponiemi et al., 2008). Other significant factors are organizational injustice, exposure to bullying, negative stereotyping, and effort–reward imbalance (Boumans et al., 2008; Heponiemi et al., 2008; Derycke et al., 2010; Suadicani et al., 2013). Some insignificant push factors in predicting early retirement are high emotion, work environment, no pleasure in work, pressure to leave, increased pressure to perform, and clearing the way for younger people (Boumans et al., 2008; Sejbaek et al., 2013; Suadicani et al., 2013; Wargo-Sugleris et al., 2018).

Financial considerations are an important pull factor that can predict early retirement decisions. It was a significant predictor among Australian nurses (Duffield et al., 2015). Similarly, financial possibility was a significant factor among Canadian registered nurses, but not among Canadian retired allied health professionals (Hewko et al., 2019). Furthermore, financial security was a significant factor among Canadian registered nurses (Blakeley and Ribeiro, 2008). Having more time to enjoy family and other things in life were significant predictors of early retirement among Canadian registered nurses (Blakeley and Ribeiro, 2008). However, having more spare time was not important among Belgian nurses (Boumans et al., 2008). Caregiving responsibility was found significant among Canadian registered nurses, but not among Canadian allied health professionals (Hewko et al., 2019). Partner retirement was also found to be a crucial factor in nurses’ decisions regarding early retirement (Blakeley and Ribeiro, 2008; Duffield et al., 2015). Other significant factors included more freedom, successful aging, and eligibility to retire (Blakeley and Ribeiro, 2008; Wargo-Sugleris et al., 2018). However, some insignificant pull factors were pension eligibility, pursuit of hobbies, positive attitudes toward retirement, and personal circumstances (Boumans et al., 2008; Hewko et al., 2019).

High work support is an anti-push factor that was found to be significant in delaying retirement among Danish hospital employees (Suadicani et al., 2013). Similarly, high supervisor support and high coworker support were also found to be significant factors in delaying early retirement among Brazilian registered nurses (de Oliveira et al., 2017). Furthermore, over-commitment was found to be a significant factor among Brazilian registered nurses, but not important among Belgian health care professionals (Derycke et al., 2010; de Oliveira et al., 2017). High job satisfaction was found to be significant among American nurse practitioners, but not among acute care registered nurses in Florida (Falk et al., 2017; Wargo-Sugleris et al., 2018). The possibility of development was significant among Belgian nurses but not among Danish employees (Boumans et al., 2008; Sejbaek et al., 2013). High quality management was found to be significant among Danish hospital employees but not among Danish employees (Sejbaek et al., 2013; Suadicani et al., 2013). Other significant anti-push factors were holding a leadership position, high work control, and being acknowledged for good work (Blakeley and Ribeiro, 2008; Suadicani et al., 2013; de Oliveira et al., 2017). Finally, some anti-push factors found insignificant in delaying early retirement were predictability, influence at work, and meaning at work (Boumans et al., 2008).

To the best of our knowledge, there are no reports on the anti-pull factors in previous research. Anti-pull factors have been studied in other fields, such as the decision-making process of young French people and Spanish people, whether to study, work, or live in another European country (Mullet et al., 2000; de Gómez Rubio et al., 2002). This study will expand on the anti-pull factors regarding early retirement decisions within the context of the Saudi medical staff.

Many studies have investigated the effects of gender differences on retirement intention. Some concluded that men tend to retire earlier than women (de Oliveira et al., 2017; Wargo-Sugleris et al., 2018), while others have established the opposite (Sejbaek et al., 2013; Peisah et al., 2017). A few studies found no significant difference between the two genders regarding early retirement intention (Heponiemi et al., 2008; Suadicani et al., 2013; Falk et al., 2017).

As far as we know, there are no studies on the factors affecting early retirement decisions among Saudi medical staff. Based on the literature review outlined above, the following five hypotheses were formulated:

H1: Push factors have a positive impact on the early retirement intention.

H2: Pull factors have a positive impact on the early retirement intention.

H3: Anti-push factors have a negative impact on the early retirement intention.

H4: Anti-pull factors have a negative impact on the early retirement intention.

H5: There is no gender difference between men and women regarding the early retirement intention.

## Materials and Methods

### Questionnaire Design

The Workers’ Retirement Motivations Inventory (WRMI) questionnaire was adopted in this study to measure the push, pull, anti-push, and anti-pull factors of early retirement decisions among the Saudi medical staff (Fouquereau et al., 2018). Each factor was measured using five questions. The participants were asked to assess each of the 20 considerations using a 10-point Likert-scale, in which 1 signified “Not important at all” while 10 signified “Very important.” Table 1 presents the 10 considerations to retire early classified into push and pull factors. Furthermore, Table 2 presents the 10 considerations to continue working until the normal retirement age, classified as anti-push and anti-pull factors (Fouquereau et al., 2018). In this study, early retirement intention was measured through a binary variable (yes or no) using the following question: “Have you ever considered retiring before your full retirement age?” (von Bonsdorff et al., 2010).

**TABLE 1:** Considerations to retire early, before the age of 60.

No.	Factor	Category
1	Feeling less motivated at work	Push
2	Feeling that the work atmosphere is not pleasant	Push
3	Having the impression that my work conditions have deteriorated	Push
4	Feeling stressed by my job	Push
5	Feeling dissatisfied with my work conditions	Push
6	Being able to spend more time with my family when I retire	Pull
7	Being able to relax when I retire	Pull
8	Being able to control my personal life better when I retire	Pull
9	To be under less pressure in general when I retire	Pull
10	Being able to spend more time with my friends when I retire	Pull

**TABLE 2:** Considerations to continue working until or after the age of 60.

No.	Factor	Category
1	Being attached to my companies	Anti-push
2	Feeling that I can still play an active role at work	Anti-Push
3	Being attached to my professional status	Anti-push
4	Still having professional ambitions	Anti-push
5	Feeling that my professional work gives me social recognition	Anti-push
6	Being afraid of losing my energy when I retire	Anti-pull
7	Being afraid of feeling depressed when I retire	Anti-pull
8	Being afraid of growing old quickly when I retire	Anti-pull
9	Being afraid of feeling lonely when I retire	Anti-pull
10	Being afraid of being bored when I retire	Anti-pull

### Population and Sample

The targeted population for this study was the Saudi medical staff aged 40 years or older. Most staff within this age class were eligible for early retirement. Data were collected using convenient sampling through an online survey distributed by a group of university students from different parts of the country. The actual response rate could not be determined because the survey was distributed online to an unknown group size.

A total of 680 responses were received in 2020. After excluding invalid responses—those who were non-Saudis (13 responses), whose age was more than 60 (8 responses), or whose age was less than 40 (438 responses)—a final sample of 221 responses was considered for analysis. Table 3 presents a summary of the demographic characteristics of the participants. The number of men was 105 (47.51%), and that of women was 116 (52.49%). Participants’ occupations were: 128 (57.92%) physicians, 27 (12.22%) nurses, and 66 (29.86%) of other occupations. Most respondents (94.57%) were married and lived with their partners, while the remaining 5.43% were single. The average number of years of service was 22.36 with a standard deviation of 6.38 years.

**TABLE 3:** Demographic characteristics of the sample: frequencies, means and standard deviations.

Variable	M (SD)	Frequency (%)
*Gender*		
Male		105 (47.51%)
Female		116 (52.49%)
*Occupation*		
Physician		128 (57.92%)
Nurse		27 (12.22%)
Other		66 (29.86%)
*Marital status*		
Living with a partner		209 (94.57%)
Single		12 (5.43%)
*Years of work experience*	22.36 (6.38)	

Table 4 shows the number and percentage of Saudi medical staff in terms of gender and occupation (Saudi Ministry of Health, 2020). Approximately 57% of the medical staff were male, while the remaining 43% were female. Moreover, 18% of the medical staff were physicians, while 36% were nurses and 46% belonged to other medical occupations. In our sample, 47.5% were men while women represented 52.5%. Furthermore, the percentages of physicians, nurses, and other occupations were 57.9, 1.2, and 29.9%, respectively.

**TABLE 4:** Population and sample distributions.

	Population (all ages)	Percentage%	Sample (40 years or older)
Total Saudi medical staff	237,382		221
Total Saudi males	136,181	57%	47.5%
Total Saudi females	101,201	43%	52.5%
Total physicians	43,581	18%	57.9%
Total nurses	84,344	36%	12.2%
Total other occupations	109,457	46%	29.9%

While we targeted Saudi medical staff members aged 40 years or older, the data are available for the entire Saudi medical population, of all ages. Even though we did not have accurate information about the target population, we ran the goodness-of-fit test to examine whether the sample had the same percentage distribution as that of the whole population. The results of the test in terms of gender are presented in Table 5. Since the *p*-value is 0.004, we concluded that the gender distribution within our sample was not similar to that of the entire population. Similarly, the goodness-of-fit test was used to examine whether the distribution of the occupation in the sample was the same as that of the entire medical population. The results in Table 6 again show that it was not, as the *p*-value was less than 0.001.

**TABLE 5:** Goodness-of-fit test for gender.

Gender	Observed N	Expected N	Residual
Male	105	126.0	–21.0
Female	116	95.0	21.0
Total	221		
Chi-square	8		
Df	1		
Asymp. Sig.	0.004		

**TABLE 6:** Goodness-of-fit test for occupation.

Occupation	Observed N	Expected N	Residual
Physician	128	39.8	88.2
Nurse	27	79.6	–52.6
Other	66	101.7	–35.7
Total	221		
Chi-square	242.877		
Df	2		
Asymp. Sig.	0.000		

Based on the above analysis, it is difficult to assess the sample’s representativeness of the target population. However, we may use the results of this study as an initial step to explore and understand the factors leading Saudi medical staff to retire early. Future research should be conducted to verify the results of this study.

### Ethical Considerations

Each participant was asked for their consent to voluntarily participate in the study. The consent form outlined the study and its objectives. Moreover, the anonymity of the participants was ensured.

## Results

Research question 1 concerning the retirement intention was answered using relative frequencies. The results indicate that more than half of the sample, 129 (58.37%) respondents, intended to retire early, before the age of 60, while the remaining 92 respondents (41.63%) planned to continue working until or after the age of 60.

Question 2 referred to the significant factors that predict the intention of the Saudi senior medical staff to retire early, while Question 3 inquired whether there was a difference between men and women in the intention to retire early. To answer these two questions, logistic regression was implemented to model the intention to retire as the dependent variable vs. the independent variables—the push, pull, anti-push, and anti-pull factors. Furthermore, three control variables were added: gender, occupation, and years of service. The gender was a binary variable with two outcomes: female (0) and male (1). Interaction terms between gender and each of the push, pull, anti-push, and anti-pull variables have also been included. The dependent variable was set as a binary variable to measure whether the respondent intended to retire early (1) or not (0).

Table 7 shows the means, standard deviations, Cronbach’s alpha, and factor loadings using confirmatory factor analysis. The Cronbach’s alpha values for the push, pull, anti-push, and anti-pull factors were 0.83, 0.89, 0.82, and 0.90, respectively, thereby indicating a high level of internal reliability. The factor loading was determined using a confirmatory factor analysis with VARIMAX rotation. The results support the validity of the questionnaire, as the loadings for each factor are above 0.50 and there was no cross-factor loading.

**TABLE 7:** Means, standard deviations, Cronbach’s alpha, and factor loading using confirmatory factor analysis.

		M	SD	Cronbach’s alpha	Push	Pull	Anti-push	Anti-pull
1	Push	6.83	2.08	0.83				
	1a	6.75	2.68		0.78	(0.02)	0.07	0.12
	1b	6.97	2.70		0.86	0.01	0.04	(0.04)
	1c	6.44	2.77		0.76	0.14	0.06	0.08
	1d	7.13	2.74		0.67	0.39	(0.01)	0.13
	1e	6.85	2.58		0.65	0.36	0.06	0.03
2	Pull	7.55	2.18	0.89				
	2a	8.21	2.41		(0.03)	0.81	0.07	0.04
	2b	7.76	2.60		0.20	0.84	(0.09)	0.07
	2c	7.60	2.62		0.08	0.88	0.02	0.08
	2d	7.60	2.60		0.27	0.80	(0.01)	0.13
	2e	6.56	2.90		0.14	0.74	0.12	0.04
3	Anti-push	7.19	1.94	0.82				
	3a	6.30	2.89		(0.01)	0.16	0.61	0.13
	3b	7.78	2.24		0.12	(0.09)	0.82	0.12
	3c	7.55	2.42		0.02	(0.01)	0.88	0.14
	3d	7.48	2.35		0.06	(0.04)	0.88	0.14
	3e	6.83	2.73		0.08	0.17	0.53	0.44
4	Anti-pull	6.02	2.50	0.90				
	4a	6.67	2.74		0.16	(0.00)	0.27	0.74
	4b	6.14	2.85		0.08	0.11	0.16	0.86
	4c	5.64	3.14		0.02	0.06	0.10	0.84
	4d	5.43	3.10		0.04	0.06	0.08	0.86
	4e	6.22	2.97		0.03	0.08	0.12	0.82

To determine the significant factors, the results of the logistic regression, using STATA 17 software, including all variables are shown in Table 8. The results show that some factors were significant, with *p*-values lower than 0.10, while others were insignificant. For example, the push factor was not statistically significant (*p* = 0.565). On the contrary, the pull, anti-push, anti-pull factors, and gender were significant. The logistic regression with backward elimination was run again to include only significant factors. The results are listed in Table 9. The pull factor had a positive sign and was significant, with a *p*-value of 0.095. However, the interaction term between the pull factor and the gender was not significant, with a *p*-value of 0.978. Furthermore, the anti-push and the interaction term between the anti-push and gender were significant with *p*-values of 0.004 and 0.012, respectively. Similarly, the anti-pull factor and the interaction between the anti-pull and gender were significant with *p*-values of 0.024 and 0.087, respectively. Finally, there was a gender difference between men and women. The latter tend to retire earlier than the former, as the sign of the variable was negative and the *p*-value was less than 0.001.

**TABLE 8:** Results of the logistics regression analysis (all independent variables).

Intention	Coefficient	Std. error	Z	P	[95% Conf. interval]	
Push	–0.086	0.150	–0.58	0.565	–0.380	0.207
Pull	0.221	0.124	1.78	0.075	–0.022	0.465
Anti-push	–0.555	0.184	–3.02	0.003	–0.915	–0.195
Anti-pull	–0.203	0.102	–1.98	0.048	–0.403	–0.002
Gender	–7.524	1.967	–3.83	0.000	–11.380	–3.670
Push by gender	0.230	0.187	1.23	0.219	–0.137	0.596
Pull by gender	–0.039	0.166	–0.23	0.815	–0.365	0.287
Anti-push by gender	0.530	0.221	2.40	0.016	0.097	0.962
Anti-pull by gender	0.219	0.148	1.48	0.138	–0.070	0.509
Physicians	0.533	0.358	1.49	0.137	–0.170	1.236
Nurses	1.042	0.575	1.81	0.070	–0.084	2.168
Year of experience	0.047	0.028	1.67	0.096	–0.008	0.102
Constant	3.824	1.620	2.36	0.018	0.649	6.998

**TABLE 9:** Results of the logistics regression analysis (only significant variables).

Intention	Coefficient	Std. error	Z	P	[95% Conf. interval]	
Gender	–6.264	1.769	–3.54	0.000	–9.730	–2.797
Pull	0.169	0.101	1.67	0.095	–0.029	0.366
Anti-pull	–0.222	0.098	–2.26	0.024	–0.415	–0.029
Anti-push	–0.498	0.175	–2.85	0.004	–0.842	–0.155
Gender by pull	–0.004	0.150	–0.03	0.978	–0.298	0.290
Gender by Anti-pull	0.244	0.143	1.71	0.087	–0.035	0.525
Gender by Anti-push	0.532	0.211	2.52	0.012	0.118	0.947
Constant	4.733	1.474	3.21	0.001	1.843	7.622

**FIGURE 1:**
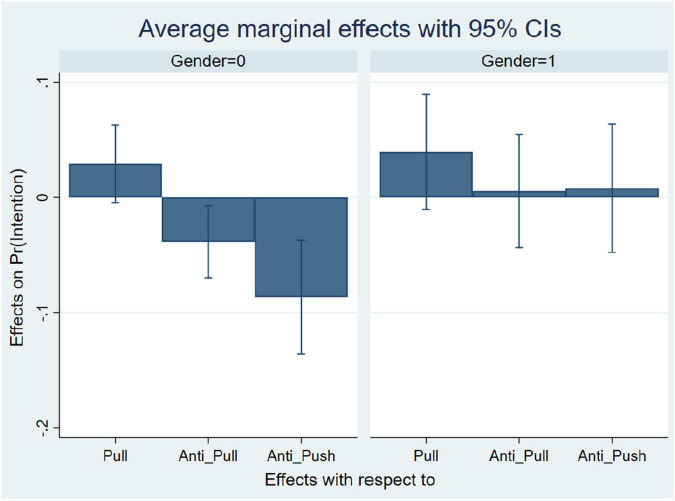
Average marginal effects (Females = 0, Males = 1).

Additional post analysis was performed where the average marginal effects of the pull, anti-push and the anti-pull factors were calculated for men and women separately. The results are shown in Table 10 and Figure 1. The average marginal effects of the pull was significant for female, with a *p*-value of 0.089, while the average marginal effects of the pull was not significant for male, with a *p*-value of 0.120. Similarly, the average marginal effects of the anti-push was significant for female, with a *p*-value of 0.001, while the average marginal effects of the anti-push was not significant for male, with a *p*-value of 0.778. Finally, the average marginal effects of the anti-pull was significant for female, with a *p*-value of 0.016, while the average marginal effects of the anti-pull was not significant for male, with a *p*-value of 0.828. Based on the previous analysis, the following can be concluded:

**TABLE 10:** Marginal effects for significant factors pull, anti-pull and anti-push.

Intention	dy/dx	Std. error	Z	P	[95% Conf. interval]	
Pull						
Females (0)	0.029	0.017	1.70	0.089	–0.004	0.062
Males (1)	0.039	0.025	1.55	0.120	–0.010	0.089
Anti-pull						
Females (0)	–0.038	0.015	–2.41	0.016	–0.069	–0.007
Males (1)	0.005	0.025	0.22	0.828	–0.043	0.054
Anti-push						
Females (0)	–0.086	0.025	–3.44	0.001	–0.135	0.037
Males (1)	0.008	0.028	0.28	0.778	–0.047	0.063

1. The push factor was not significant. Therefore, Hypothesis 1 is not supported.
2. The pull factor was significant as the *p*-value is 0.095. However, based on the analysis of marginal effect, the pull factor was significant for women with a *p*-value of 0.089 while it is not significant for men as the *p*-value is 0.12. Therefore, Hypothesis 2 is partially supported.
3. The anti-push factor was significant with a *p*-value of 0.004. However, post analysis showed that it is significant for women with a *p*-value of 0.001 but not for men as the *p*-value is 0.778. Therefore, hypothesis 3 is partially supported.
4. The anti-pull factor was significant with a *p*-value of 0.024. However, post analysis showed that it is significant for women with a *p*-value of 0.016 but not for men as the *p*-value is 0.828. Therefore, hypothesis 4 is partially supported.
5. Hypothesis 5 is supported as there is a significant difference between women and men in the intention to retire early with a *p*-value less than 0.001. Women tend to retire earlier than men.

## Discussion

This study answers three main questions. First, around 58% of the Saudi medical staff intends to retire before the retirement age of 60, while around 42% intend to continue working until or after the retirement age. Based on the existing ratio of Saudis in the medical sector (53%), it seems that the problem of the low proportion of Saudis will persist for some time unless new Saudi graduates enter the medical sector. Additionally, lowering the proportion of early retirement intention among the current Saudi medical staff will help overcome this problem.

The second question addressed the significant factors that predict early retirement intention. These are the pull, anti-push, and anti-pull factors. Therefore, keeping the medical staff for a longer employment period requires a closer look at these factors. In fact, Beehr (1986) classified the factors leading to retirement into two main categories: personal factors—such as health and economic well-being—and environmental factors—including work factors, such as being tired of work, and non-work factors, such as leisure time. The model proposed that some factors may push employees to retire or pull them to stay in the workplace. In this study, the pull factors included items such as having more time to spend with family and friends, relaxing after retirement, having better control over personal life, and being less pressured after retirement. It is clear that the medical staff needs more time to pursue other enjoyable activities. Providing flexible working hours or reducing their number might be a solution for sufficient leisure time (Blakeley and Ribeiro, 2008). The anti-push factors were found to be significant and included items such as feeling attached to a person’s company, playing active roles at work, being attached to professional status, having professional ambitions, and feeling that professional work gives social recognition. It is clear that being a medical professional is perceived as prestigious. Saudi medical staff would prefer to continue working as long as they continue to receive this recognition of their efforts and talents. Moreover, empowering senior medical staff to have more control over their duties could positively affect the work environment, thereby delaying early retirement (Blakeley and Ribeiro, 2008; Heponiemi et al., 2008; Duffield et al., 2015).

The anti-pull factors included items such as losing energy after retirement, feeling depressed, growing older faster, feeling lonely, and being bored after retirement. It is possible to carry out an awareness program on these consequences. This program could focus on emphasizing positive rewards and providing precautions about possible negative consequences. We believe that this move could positively affect early retirement and delay it.

Finally, the study addressed a third question regarding gender differences in the intention of early retirement. The results show that women intend to retire earlier than men. This is explained by the fact that women in the traditional Saudi society play an important role in caring for family members. This result is supported by many studies conducted in other countries (Boumans et al., 2008; Sejbaek et al., 2013; Peisah et al., 2017).

## Conclusion

Although it is important to train and recruit new medical national staff to meet the increasing demand for medical services in Saudi Arabia, it is equally important to understand the factors that contribute to retain the existing experienced staff. This study investigated the relationship between Fouquereau’s push, pull, anti-push, and anti-pull factors and early retirement intention among Saudi medical staff. The significant factors found were the pull, anti-push, and anti-pull. Moreover, women seem to intend to retire earlier than men.

This study contributes to the literature by implementing Fouquereau’s framework to the early retirement intention of the medical staff in Saudi Arabia (Fouquereau et al., 2018). However, this study had some limitations. First, it focused on the broad factors that lead to early retirement. Since there is no published work on the subject within the Saudi context, this study was designed to obtain an overall view of such factors. However, it lacks a deep analysis of each factor. Future research might consider other issues, such as focusing on the anti-pull factor, which was found to be significant in this study and has not been investigated thoroughly in the literature. Another limitation is that the response variable, retirement intentions, and the explanatory variables were all included in the same survey. It may lead to a single source bias as recipients might answer the questions in a way that justifies their intention to retire early. Furthermore, although the information obtained is useful in linking the intention of early retirement with the used framework, it is more informative to conduct a longitudinal study to better understand the relationship between the intention to retire and the actual retirement decision as well as the factors contributing to this decision over time. Moreover, the number of valid responses received in this study was only 221, and this sample may not fully represent the target population, which might have limited the generalization of the results to the whole population. Finally, future studies should obtain more information to better extend the results to the population and should also account for any bias in the estimated effects using more information about employees’ history and wages (Böckerman et al., 2012).

The practical implication of this study is that it helps retain existing Saudi senior medical staff longer in the profession. Some practices can be adopted to achieve this goal. For example, the pull factors were found to be significant, thus indicating that senior medical staff are looking for more free time to spend with their friends and family. Therefore, having flexible work hours and/or providing shorter shifts or part-time jobs to senior medical staff could create a balance between providing medical services while enjoying their lives. Additionally, the senior staff seem to suffer from workplace pressure, and they intend to retire early to reduce their exposure to it. Although it is very difficult to eliminate pressure completely, the senior staff may be given less workload and reduced working hours. Furthermore, the anti-push factors were also found to be significant, thereby indicating that the medical staff would not mind staying longer in their profession as long as the attachment to the current work environment is rewarding. Therefore, more emphasis should be placed on acknowledging and valuing their contributions. Moreover, more autonomy at the workplace and providing the opportunity to play an active role will help them stay longer in the profession (Blakeley and Ribeiro, 2008; Boumans et al., 2008). Finally, the anti-pull factors were also significant, thus indicating that the medical staff may delay retirement decisions fearing that they may grow older quickly, lose energy, or get depressed after early retirement. Providing awareness programs about the negative consequences of early retirement may help delay the retirement decisions.

## Data Availability Statement

The raw data supporting the conclusions of this article is available in the following 10.6084/m9.figshare.17126234.v1.

## Ethics Statement

Ethical review and approval was not required for the study on human participants in accordance with the local legislation and institutional requirements. The patients/participants provided their written informed consent to participate in this study.

## Author Contributions

TA and MA: conceptualization. MA and AA: methodology, software, validation, and resources. TA, MA, and AA: formal analysis. MA: writing—original draft preparation. TA and AA writing—review and editing. TA: supervision. All authors contributed to the article and approved the submitted version.

## Conflict of Interest

The authors declare that the research was conducted in the absence of any commercial or financial relationships that could be construed as a potential conflict of interest.

## Publisher’s Note

All claims expressed in this article are solely those of the authors and do not necessarily represent those of their affiliated organizations, or those of the publisher, the editors and the reviewers. Any product that may be evaluated in this article, or claim that may be made by its manufacturer, is not guaranteed or endorsed by the publisher.
